# Impact of Geriatric Events on Clinical Outcomes and Resource Utilization of Acute Coronary Syndrome Hospitalizations

**DOI:** 10.7759/cureus.35319

**Published:** 2023-02-22

**Authors:** Victory Okpujie, Fidelis Uwumiro, Osasumwen F Osemwota, Ruth Pius, Esere Obodo, Grace D Ogunkoya, Olawale Abesin, Ayodeji Ilelaboye, Michael M Bojerenu, Assumpta Obidike

**Affiliations:** 1 Internal Medicine, University of Benin Teaching Hospital, Benin City, NGA; 2 Internal Medicine, Department of Health Sciences & Social Work, Western Illinois University, Macomb, USA; 3 Internal Medicine, College of Medicine, University of Lagos, Lagos, NGA; 4 Internal Medicine, Federal Medical Centre Abuja, Abuja, NGA; 5 Internal Medicine, Lagos State University College of Medicine, Lagos, NGA; 6 Internal Medicine, Royal Cornwall Hospital NHS (National Health Service) Trust, Truro, GBR; 7 Internal Medicine, Ladoke Akintola University of Technology, Ogbomoso, NGA; 8 Internal Medicine, St. Barnabas Hospital, SBH (St Barnabas Hospital) Health System, New York City, USA; 9 Internal Medicine, Chukwuemeka Odumegwu Ojukwu University Teaching Hospital, Awka, NGA

**Keywords:** older adult, delirium, myocardial infarction, acute coronary syndrome, geriatric events

## Abstract

Background

The effect of geriatric events (GEs) on outcomes of acute coronary syndrome (ACS) admissions is poorly understood. We evaluated the prevalence and impact of GEs on clinical outcomes and resource utilization of older patients admitted with ACS.

Methods

Using the 2018 National (Nationwide) Inpatient Sample, we analyzed all elective hospitalizations for ACS in older adults (age ≥ 65 years) and a younger reference group (age 55-64). Nationally-weighted descriptive statistics were generated for GEs based on ACS subtypes. Multivariate logistic regression models controlling for comorbidities, frailty, patient procedure, and hospital-level variables were used to estimate the association of age with GEs and GEs with outcomes.

Results

Out of 403,760 admissions analyzed, 71.9% occurred in older adults (≥65 years). The overall rate of any GE in older adults with ACS was 3.4%. With advancing age, the number of GEs was found to significantly increase (p<0.001). After adjustments, having any GE was found to have a significant impact on mortality (adjusted OR (AOR): 1.32; 95%CI: 1.15-1.54; p < 0.001), post-myocardial infarction (MI) complications (AOR: 1.53; 95%CI: 1.36-1.71; p < 0.001), prolonged hospital stays (AOR: 2.97; 95%CI: 2.56-3.30; p < 0.001), and non-home (acute care and skilled nursing home) discharge (AOR: 1.68; 95%CI: 1.53-1.85; p < 0.001). The occurrence of GEs was also associated with a substantial increase in total hospitalization costs with a mean increase of $48,325.22 ± $5,539 (p < 0.001). A dose-response relationship was established between GEs and all outcomes. Limitations of the study included the use of retrospective data and an administrative database.

Conclusion

Geriatric events were found to significantly worsen outcomes for older adults with ACS. There is, therefore, a need for increased awareness and effective management of GEs in older adults to improve their health outcomes and reduce the burden on the healthcare system.

## Introduction

The aging population (≥ 65 years) in the United States is projected to increase by 50% by 2050 [1]. With aging, a decline in multiple physiological processes is observed and has been recognized as a significant contributor to cardiovascular disease [2]. The literature abounds with evidence linking advanced age to inferior clinical outcomes in various patient groups [3,4]. Additionally, elderly patients face unique risks of geriatric events (GEs), such as delirium and dehydration, which have also been linked to poor clinical outcomes in previous research [5].

Approximately 60% of hospital admissions for acute coronary syndrome (ACS) are for individuals over 65 years of age, and about 85% of ACS-related deaths occur in this population [6]. Despite the broad knowledge about ACS and GEs, the relationship between GEs and the outcomes of ACS remains complex and poorly understood due to the confounding effects of frailty and other comorbidities. Therefore, understanding the impact of geriatric events on the outcomes of ACS is important for improving the care and management of the older patient population. Using national data, this study aims to examine the prevalence of GEs and assess the relationship between GEs and clinical outcomes among patients hospitalized for ACS while adjusting for the effects of comorbidity and physical frailty.

## Materials and methods

Data source

The 2018 National (Nationwide) Inpatient Sample (NIS) was queried for all elective hospitalizations related to ACS. For comparison, the study population included elderly persons (65 years) and a younger age group (55-64 years). Although the descriptive analysis focused on older people, a younger reference group was included to investigate the relationship between age and GEs.

Outcome variables

Patient variables included age category (55-64 years; 65-74 years; ≥75 years), race or ethnicity, sex, median income in the patient’s Zone Improvement Plan (ZIP) code, and the Charlson comorbidity index (CCI). Procedures and diagnoses variables were defined using the International Classification of Diseases, 10th revision, Clinical Modification, and Procedure Coding System (ICD-10-CM/PCS). Hospital variables included hospital bed size (small, medium, and large), location and teaching status (urban teaching, urban non-teaching, and rural), and the expected primary payer (Medicare, Medicaid, private including Health Maintenance Organization (HMO), and self-pay). Geriatric events (delirium, dehydration, falls or fragility fractures, failure to thrive, and pressure ulcers) were defined using a combination of the relevant ICD-10-CM/PCS codes and statistical commands. Clinical outcomes of interest included sudden cardiac death, all-cause mortality, post-myocardial infarction (MI) complications (arrhythmias, cardiogenic shock, refractory cardiac arrest, atrial and ventricular septal defects, hemopericardium, ventricular wall ruptures, and thrombosis), prolonged length of stay (LOS), and non-home discharge (which included all discharges to skilled nursing facilities (SNF) and acute care hospitals). Prolonged LOS was defined as a diagnosis-specific LOS in the top decile.

Statistical analysis

Statistical analysis was performed using Stata® version 17 (StataCorp LLC, College Station, Texas). The p-value was determined using two-tailed tests with a significance level of 0.05. The analyses were based on weighted samples to provide national estimates. The relationship between age and GEs and the impact of GEs on clinical outcomes and LOS were examined using multivariate logistic regression models. The models were adjusted for patient characteristics (age, sex, race, national quartile for median household income of patient's ZIP Code, and insurance status), Charlson comorbidity index, frailty (which was defined using the Johns Hopkins Adjusted Clinical Groups frailty-defining clusters), procedures, time to revascularization, and hospital factors (hospital location/teaching status, and hospital bed size). The research also explored the potential linear relationship between the number of GEs and clinical outcomes. The national-level descriptive statistics were generated for GEs based on age group (55-64 years, 65-74 years, ≥75 years) and diagnosis. Using chi-squared statistical tests, the prevalence of GEs was compared across age groups and diagnoses. Categorical and continuous variables are reported as proportions or mean with standard deviation. Regression outcomes are reported as adjusted odds ratios (AORs) with 95% confidence intervals (CIs).

Ethical considerations

This research endeavor did not require institutional review board approval owing to the fact that it relied exclusively on publicly accessible retrospective data procured from the NIS database. Since 2012, this database has been purged of any patient- and hospital-level identification information.

## Results

Within the total study population of individuals aged 55 years or older, the total number of hospitalizations due to ACS was 403,760, of which a nationally-weighted 71.9% (290,605) occurred in older adults aged 65 or older. When considering solely admissions for older adults, the vast majority, 94.6% (274,975), were admitted with non-ST-elevation myocardial infarction (NSTEMI), while STEMI represented 3.2% (9,360), unstable angina 2% (5,750), and repeat MI 0.2% (520). A total of 0.62% (1,798) of patients underwent revascularization procedures, either by means of coronary artery bypass grafting (CABG) or percutaneous coronary intervention (PCI).

Analysis of the overall clinical outcomes among the older adult population revealed the following results: a mortality rate of 5.1% (14,859 cases), post-MI complications in 10.4% (30,222 cases), a prolonged LOS in 27.5% (79,916 cases), and a non-home discharge rate of 25.9% (75,267 cases). The incidence of sudden cardiac death was 1.5% while the mean time to revascularization was 1.2 ± 0.1 days.

The overall incidence of any GE among the entire study cohort was found to be 3.4% (13,728 hospitalizations), and this rate was observed to increase with increasing age categories, with the highest rate of 4.8% observed among individuals aged 75 years or older (55-64 years: 1.9%; 65-74 years: 3.1%; ≥75 years: 4.8%; p<0.001). A similar trend was observed for each individual GE, with the incidence increasing with age (p < 0.001). Among older adults, a minimum of 4.2% (12,205) experienced at least one GE. The majority of these patients experienced one GE (90.4%), while 4.3% experienced two GEs, and 5.3% had three or more GEs. The prevalence of GEs varied depending on the diagnosis, with the lowest incidence (0.9%) observed for unstable angina, and the highest incidence (4.2%) observed for repeat MI (p < 0.001) (Table 1). The most common GE overall among older adults was found to be dehydration at 1.6%.

**Table 1:** Proportions of geriatric events by diagnoses in older adults (≥ 65 years) ^1^Any geriatric event does not equate to the sum of all possible geriatric events, as individuals may experience multiple types of geriatric events simultaneously ^2^All frequencies and proportions are nationally weighted. NSTEMI = non-ST-elevation myocardial infarction; STEMI = ST-elevation myocardial infarction; ST-elevation myocardial infarction

Geriatric Event	Total study population (n=403,760)		Older adults (n=290,605)		NSTEMI (n=274,975)		STEMI (n 9,360)		Unstable Angina (n=5,750)		Repeat MI (n=520)	
	No.^2^	%	No.	%	No.	%	No.	%	No.	%	No.	%
Any geriatric event^1^	13,727	3.4	11,624	4.0	8249	3.0	271	2.9	52	0.9	22	4.2
Delirium	6,056	1.5	3,777	1.3	2,502	0.9	84	0.9	7	0.1	7	1.4
Dehydration	4,199	1.0	4,649	1.6	3,849	1.4	102	1.1	4,670	23	0.4	1.5
Falls/fractures	1,656	0.4	1,046	0.4	846	0.3	113	1.2	80	1.4	7	1.4
Failure to thrive	4,441	1.1	3,487	1.2	2,749	1.0	84	0.9	29	0.5	10	1.9
Pressure ulcers	10	0.003	9	0.003	5	0.002	4	0.04	0	0	0	0.2

**Figure 1:**
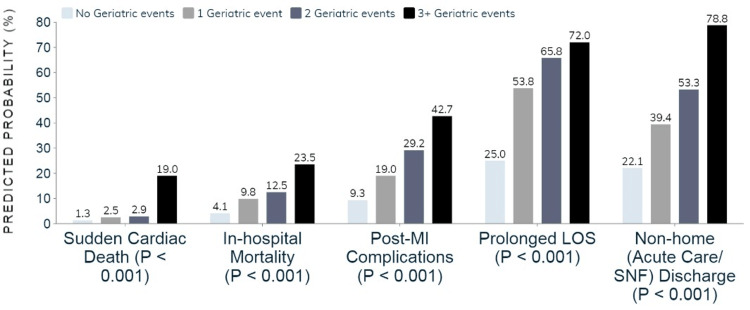
Adjusted probabilities of clinical outcomes by the number of geriatric events Post-MI complications include arrhythmias, cardiogenic shock, refractory cardiac arrest, atrial and ventricular septal defects, hemopericardium, ventricular wall ruptures, and thrombosis. Prolonged LOS was defined as a diagnosis-specific LOS in the top decile. LOS = length of hospital stay; SNF = skilled nursing facility; MI = myocardial infarction

After controlling for patient and hospital-level variables, the presence of GEs was found to be correlated with an elevated likelihood of mortality (AOR: 1.32; 95%CI: 1.15-1.54; p < 0.001), post-MI complications (AOR: 1.53; 95%CI: 1.36-1.71; p < 0.001), prolonged LOS (AOR: 2.97; 95%CI: 2.56-3.30; p < 0.001), and non-home (acute care and skilled nursing home) discharges (AOR: 1.68; 95%CI: 1.53-1.85; p < 0.001). The occurrence of GEs was also associated with higher total hospitalization costs (mean increase of $48,325.22 ± $5,539; p < 0.001). A dose-response relationship was identified between GEs and all outcomes, such that with each additional GE, the predicted probability of unfavorable outcomes increased in a linear manner (Figure 1).

## Discussion

Old age is one of the strongest predictors of poor outcomes in patients with ACS [7]. The results of this study provide important insights into the incidence and clinical outcomes of geriatric events among older adults hospitalized due to ACS. This is the first study, to our knowledge, to demonstrate how common GEs are in the setting of ACS. The study found that the overall incidence of GEs among the study cohort was 3.4%, and this rate increased with increasing age categories. In comparison, previous research has found a much higher overall rate of GEs (23.1%) in a cohort of patients undergoing procedures [8]. This suggests that the overall rate of GEs in the wider older patient population may be higher than this study suggests.

Geriatric events can occur in older patients due to a variety of reasons, including (i) Chronic health conditions: Chronic conditions commonly associated with aging, such as heart disease, diabetes, and lung disease, can increase the risk of geriatric events; (ii) Cognitive impairment: Dementia and other forms of cognitive decline can increase the risk of events such as falls, failure to thrive, and dehydration by impairing the ability to make decisions, follow instructions, and perform daily activities [9], (iii) Medication use: The use of multiple medications, referred to as polypharmacy, can increase the risk of adverse drug reactions and interactions, which can lead to geriatric events such as delirium [10], (IV) Physical frailty: Physical frailty, characterized by weakness, decreased endurance, and reduced mobility can increase the risk of falls and other types of injury [11], and (v) Social isolation: Loneliness and social isolation can increase the risk of depression, anxiety, and other mental health problems, which can increase the risk of geriatric events [12]. These factors often have a synergistic effect, emphasizing the importance for healthcare providers to consider multiple risk factors when caring for older patients with ACS.

Strategies for preventing GEs and mitigating their risk factors have been the focus of numerous research studies. Preventive home visits have been found to be effective in reducing falls among community-dwelling older adults [13]. In the inpatient setting, consistent use of bed/chair alarms for patients at risk of falling has demonstrated significant efficacy in preventing falls [14]. The use of heel suspension devices and multi-layered soft silicone foam dressings has also been reported to effectively prevent sacral and heel pressure ulcers in critically ill patients [15,16]. Several delirium prevention methods have been linked to substantial reductions in the incidence of in-hospital delirium [17,18]. Additionally, a collaborative care model and built environment interventions, such as hospital redesign, have been demonstrated to yield favorable outcomes for elderly patients with cognitive impairment [19,20].

This study had some limitations. First, the NIS, as an administrative database, is susceptible to variations in local coding practices. This could result in an underestimation of the occurrence of GEs, and thus, the actual prevalence of GEs may be higher than reported in this study. Furthermore, the NIS has limitations in providing comprehensive clinical data, including the type of admission, the LOS in a skilled nursing facility, medications administered, readmission rates, and intensity of care. Lastly, the use of retrospective data may not reflect current prevalence rates and does not provide information on patient outcomes beyond the year of the claim. Despite these limitations, the findings of the index study offer crucial insight into the impact of GEs on patient outcomes and provide a compelling rationale for the implementation of more robust GE prevention initiatives in geriatric cardiology.

## Conclusions

The study found that independent of comorbidities and frailty, geriatric events are important predictors of morbidity and mortality in older patients with ACS. The findings of this study highlight the need for healthcare providers to be aware of and proactive in preventing GEs in the care of older adults with ACS to improve outcomes and reduce admission costs. Further research is needed to explore the best strategies for identifying and managing GEs in this patient population.

## References

[1] Vincent GK, Velkoff VA (2023). U.S. Census Bureau. U.S. Population Projections: 2010 to 2050. The Next Four Decades: The Older Population in the United States: 2010 to 2050.

[2] North BJ, Sinclair DA (2012). The intersection between aging and cardiovascular disease. Circ Res.

[3] Wood B, Lee CR, Mulrenin IR, Gower MN, Rossi JS, Weck KE, Stouffer GA (2021). Effects of aging on clinical outcomes in patients receiving genotype-guided P2Y12 inhibitor selection after percutaneous coronary intervention. Pharmacotherapy.

[4] Jaul E, Barron J (2017). Age-related diseases and clinical and public health implications for the 85 years old and over population. Front Public Health.

[5] Dworsky JQ, Shellito AD, Childers CP (2021). Association of geriatric events with perioperative outcomes after elective inpatient surgery. J Surg Res.

[6] Dai X, Busby-Whitehead J, Alexander KP (2016). Acute coronary syndrome in the older adults. J Geriatr Cardiol.

[7] Montilla Padilla I, Martín-Asenjo R, Bueno H (2017). Management of acute coronary syndromes in geriatric patients. Heart Lung Circ.

[8] Dworsky JQ, Childers CP, Copeland T, Maggard-Gibbons M, Tan HJ, Saliba D, Russell MM (2019). Geriatric events among older adults undergoing non-elective surgery are associated with poor outcomes. Am Surg.

[9] van Doorn C, Gruber-Baldini AL, Zimmerman S (2003). Dementia as a risk factor for falls and fall injuries among nursing home residents. J Am Geriatr Soc.

[10] Hein C, Forgues A, Piau A, Sommet A, Vellas B, Nourhashémi F (2014). Impact of polypharmacy on occurrence of delirium in elderly emergency patients. J Am Med Dir Assoc.

[11] Cheng MH, Chang SF (2017). Frailty as a risk factor for falls among community dwelling people: evidence from a meta-analysis. J Nurs Scholarsh.

[12] Freedman A, Nicolle J (2020). Social isolation and loneliness: the new geriatric giants: approach for primary care. Can Fam Physician.

[13] Luck T, Motzek T, Luppa M (2013). Effectiveness of preventive home visits in reducing the risk of falls in old age: a randomized controlled trial. Clin Interv Aging.

[14] Melin CM (2018). Reducing falls in the inpatient hospital setting. Int J Evid Based Healthc.

[15] Santamaria N, Gerdtz M, Sage S (2015). A randomised controlled trial of the effectiveness of soft silicone multi-layered foam dressings in the prevention of sacral and heel pressure ulcers in trauma and critically ill patients: the border trial. Int Wound J.

[16] Bååth C, Engström M, Gunningberg L, Muntlin Athlin Å (2016). Prevention of heel pressure ulcers among older patients--from ambulance care to hospital discharge: a multi-centre randomized controlled trial. Appl Nurs Res.

[17] Ghaeli P, Shahhatami F, Mojtahed Zade M, Mohammadi M, Arbabi M (2018). Preventive intervention to prevent delirium in patients hospitalized in intensive care unit. Iran J Psychiatry.

[18] Fong TG, Tulebaev SR, Inouye SK (2009). Delirium in elderly adults: diagnosis, prevention and treatment. Nat Rev Neurol.

[19] Grey T, Fleming R, Goodenough BJ, Xidous D, Möhler R, O'Neill D (2019). Hospital design for older people with cognitive impairment including dementia and delirium: supporting inpatients and accompanying persons. Cochrane Database Syst Rev.

[20] Nikelski A, Keller A, Schumacher-Schönert F (2019). Supporting elderly people with cognitive impairment during and after hospital stays with intersectoral care management: study protocol for a randomized controlled trial. Trials.

